# Supplementary material for: Evolutionary plasticity of SH3 domain binding by Nef proteins of the HIV-1/SIVcpz lentiviral lineage
Source: PLoS Pathog. 2021 Nov 15;17(11):e1009728. doi: 10.1371/journal.ppat.1009728 (PMC8629392; doi:10.1371/journal.ppat.1009728)
Supplement: S1 Fig — Shown are the raw data for the histograms in Fig 1C. (PDF) [file ppat.1009728.s001.pdf]

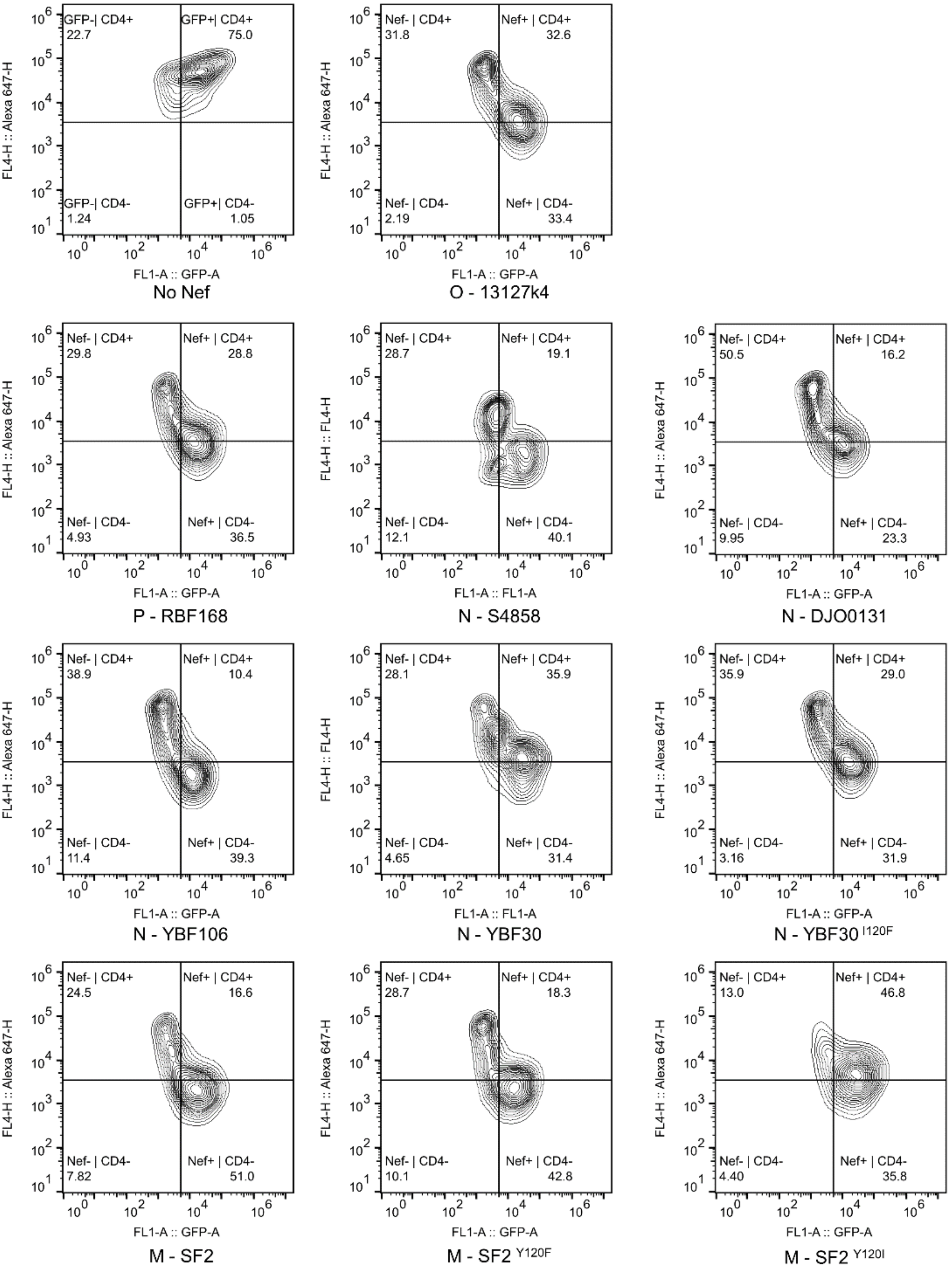

S1 Fig. Contour plots illustrating cell surface CD4 expression and HIV-1 Nef transduction (GFP) levels. Shown are the raw data for the histograms in Fig 1C.
