# Supplementary material for: Evolutionary plasticity of SH3 domain binding by Nef proteins of the HIV-1/SIVcpz lentiviral lineage
Source: PLoS Pathog. 2021 Nov 15;17(11):e1009728. doi: 10.1371/journal.ppat.1009728 (PMC8629392; doi:10.1371/journal.ppat.1009728)
Supplement: S4 Fig — Analysis of the MD trajectories show that in the wild type complex the stacked Tyr-Arg-Trp π-cation-π interaction remains stable, as interpreted from Arg to Tyr and Arg to Trp side chain distances, which show little fluctuations around their average values, 4.2 Å (A) and 3.5 Å (B). In (C) is shown a snapshot from the wild type complex simulation in which these distances are close to their average values. In the double mutant the Arg to Trp distance is on the average about 0.5 Å longer than that in the wild type complex (E), but also remains stable around its average value, 4.0 Å. If 6 Å is taken as the maximum distance for a cation-π interaction [47] the observed distances are well within the limit. Likewise, hydrophobic contacts to and between Met and Ile on the other side of the Arg plane remain relatively stable. Non-bonded contacts are likely to be bolstered by Met sulfur [48]. Ile and Arg sidechains are on average 4.4 Å apart (D). Met to Arg (F) and Ile to Met (G) distances show more variation, but are for the majority of time close to about 4.6 and 3.9 Å. In (H) is shown a snapshot from the double mutant complex simulation in which these distances are close to their average values. A salt bridge between Arg and Asp side chains is present in 96% (WT) and 93% (double mutant) of the simulation frames. MD simulations in explicit solvent were performed with AMBER 20 [24] using the ff14SB force field. The WT and A83M/Y120I complexes were placed in a cubic box with a minimum solute-box distance of 10 Å, and solvated with TIP3P water molecules. Six sodium ions were added to neutralize the system. After minimization, heating and equilibration of the system, the production 100-ns MD simulations were performed with periodic boundary conditions at 300 K. The temperature was maintained by using the Langevin thermostat, whereas the pressure was kept at 1 bar using the Berendsen barostat [49]. The time step was set to 2 fs. Long-range electrostatic interactions were treated [file ppat.1009728.s004.pdf]

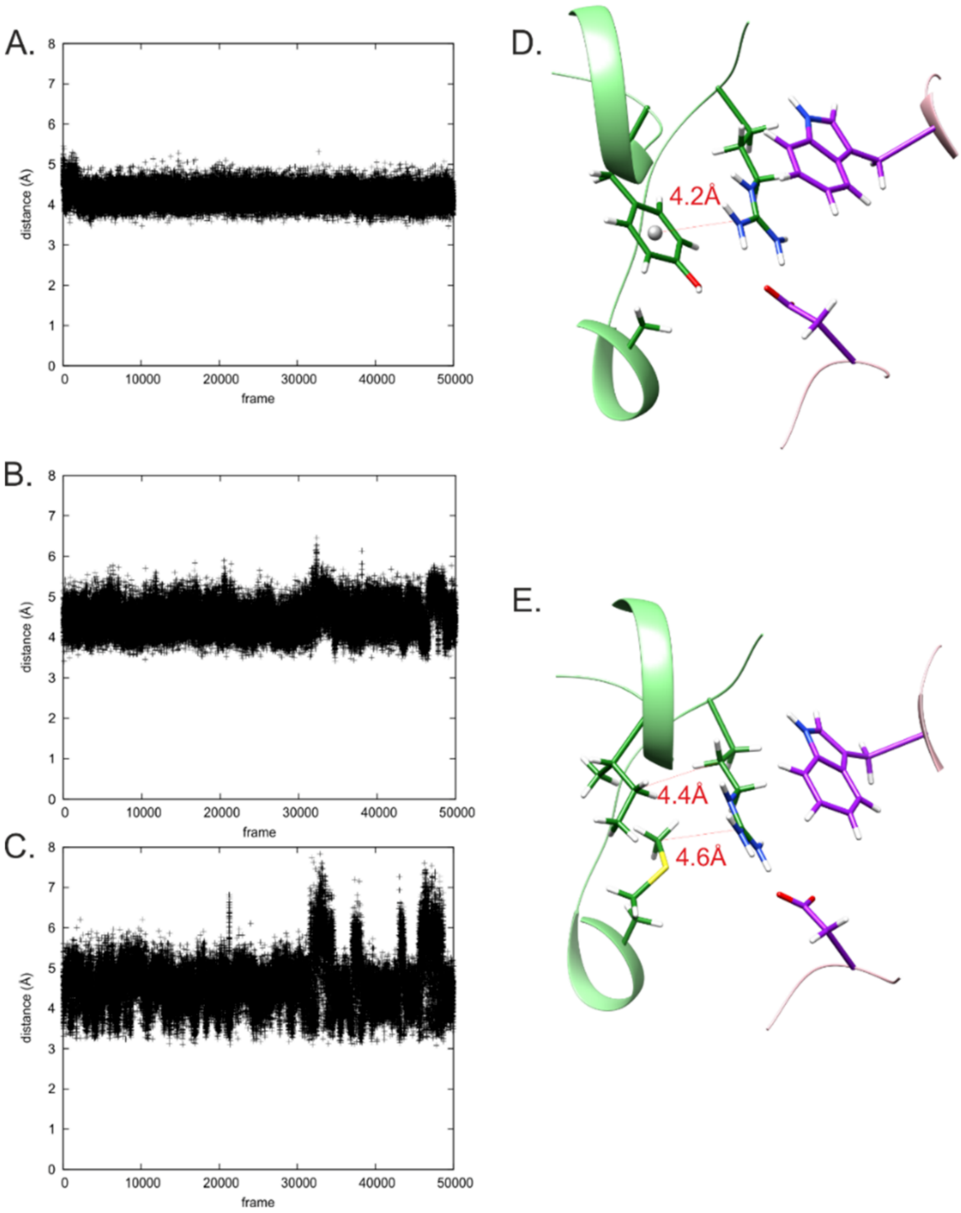

S4 Fig. One-hundred ns all-atom molecular dynamics simulations show that stabilization of R77 side chain provided by the R-clamp in the wild-type complex could be reproduced by Met, Ile and Trp in the A83M/Y120I double mutant. Analysis of the MD trajectories show that in the wild type complex the stacked Tyr-Arg-Trp π-cation-π interaction remains stable, as interpreted from Arg to Tyr and Arg to Trp side chain distances, which show little fluctuations around their average values, 4.2 Å **(A)** and 3.5 Å **(B)**. In **(C)** is shown a snapshot from the wild type complex simulation in which these distances are close to their average values. In the double mutant the Arg to Trp distance is on the average about 0.5 Å longer than that in the wild type complex **(E)**, but also remains stable around its average value, 4.0 Å. If 6 Å is taken as the maximum distance for a cation-π interaction [1] the observed distances are well within the limit. Likewise, hydrophobic contacts to and between Met and Ile on the other side of the Arg plane remain relatively stable. Non-bonded contacts are likely to be bolstered by Met sulfur [2]. Ile and Arg sidechains are on average 4.4 Å

apart **(D)**. Met to Arg **(F)** and Ile to Met **(G)** distances show more variation, but are for the majority of time close to about 4.6 and 3.9 Å. In **(H)** is shown a snapshot from the double mutant complex simulation in which these distances are close to their average values. A salt bridge between Arg and Asp side chains is present in 96 % (WT) and 93% (double mutant) of the simulation frames. MD simulations in explicit solvent were performed with AMBER 20 [3] using the ff14SB force field. The WT and A83M/Y120I complexes were placed in a cubic box with a minimum solute-box distance of 10 Å, and solvated with TIP3P water molecules. Six sodium ions were added to neutralize the system. After minimization, heating and equilibration of the system, the production 100-ns MD simulations were performed with periodic boundary conditions at 300 K. The temperature was maintained by using the Langevin thermostat, whereas the pressure was kept at 1 bar using the Berendsen barostat [4]. The time step was set to 2 fs. Long-range electrostatic interactions were treated using the Particle Mesh Ewald method [5] with a cut-off of 10 Å. Bond lengths involving hydrogen atoms were constrained by SHAKE [6]. Analyses of the trajectories were carried out with CPPTRAJ [7].

REFERENCES

1. Gallivan JP, Dougherty DA. Cation-pi interactions in structural biology. Proc Natl Acad Sci U S A. 1999;96(17):9459-64. Epub 1999/08/18. doi: 10.1073/pnas.96.17.9459. PubMed PMID: 10449714.

2. Gómez-Tamayo JC, Cordomí A, Olivella M, Mayol E, Fourmy D, Pardo L. Analysis of the interactions of sulfur-containing amino acids in membrane proteins. Protein Sci. 2016;25(8):1517-24. Epub 2016/05/31. doi: 10.1002/pro.2955. PubMed PMID: 27240306.

3. Case DA, Belfon K, Ben-Shalom IY, Brozell SR, Cerutti DS, Cheatham TEI, et al. AMBER 2020: University of California, San Francisco; 2020.

4. Berendsen HJC, Postma JPM, van Gunsteren WF, DiNola A, Haak JR. Molecular dynamics with coupling to an external bath.. 1984;81:3684–90. doi: J Phys Chem 10.1063/1.448118.

5. Darden T, York D, Pedersen L. Particle mesh Ewald: an N·log(N) method for Ewald sums in large systems. J Chem Phys. 1993;98:10089–92. doi: 10.1063/1.464397.

6. Ryckaert J-P, Ciccotti G, Berendsen HJC. Numerical integration of the cartesian equations of motion of a system with constraints: molecular dynamics of n-alkanes. J Comput Phys. 1977;23:327-41. doi: 10.1016/0021-9991(77)90098-5.

7. Roe DR, Cheatham TE, 3rd. PTRAJ and CPPTRAJ: Software for Processing and Analysis of Molecular Dynamics Trajectory Data. J Chem Theory Comput. 2013;9(7):3084-95. Epub 2013/07/09. doi: 10.1021/ct400341p. PubMed PMID: 26583988.
