# Supplementary material for: Evolutionary plasticity of SH3 domain binding by Nef proteins of the HIV-1/SIVcpz lentiviral lineage
Source: PLoS Pathog. 2021 Nov 15;17(11):e1009728. doi: 10.1371/journal.ppat.1009728 (PMC8629392; doi:10.1371/journal.ppat.1009728)
Supplement: S5 Fig — HEK293 cells were transfected with Myc-tagged wild-type HIV-1 M SF2 Nef (WT) or its mutants including Y120I, A83L-Y120I, P76A+P78A (AxxA), A83Q, and A83Q-Y120I) together with biotin acceptor domain-tagged SH3 mutated Hck-p59 variants Hck-A1 (A.) or Hck-B6 (B.). Similarly, YBF30 or its R-clamp gain-of-function mutant Q83M were co-expressed with biotin acceptor domain-tagged wild-type Hck-p59 or its SH3 mutant Hck-B6. (C.). The Nef-contacting RT-loop residues centered around the critical Hck SH3 amino acid 13 (indicated in bold) are shown on top of these Figs. Lysates of the transfected cells were subjected to anti-Myc immunoprecipitation followed by Western blotting analysis of the immune complexes using labeled streptavidin (top panels). Equal Hck and Nef expression in the total lysates was confirmed by labeled streptavidin (Hck) (middle panels) or an anti-Myc (Nef) antibody (bottom panels). (PDF) [file ppat.1009728.s005.pdf]

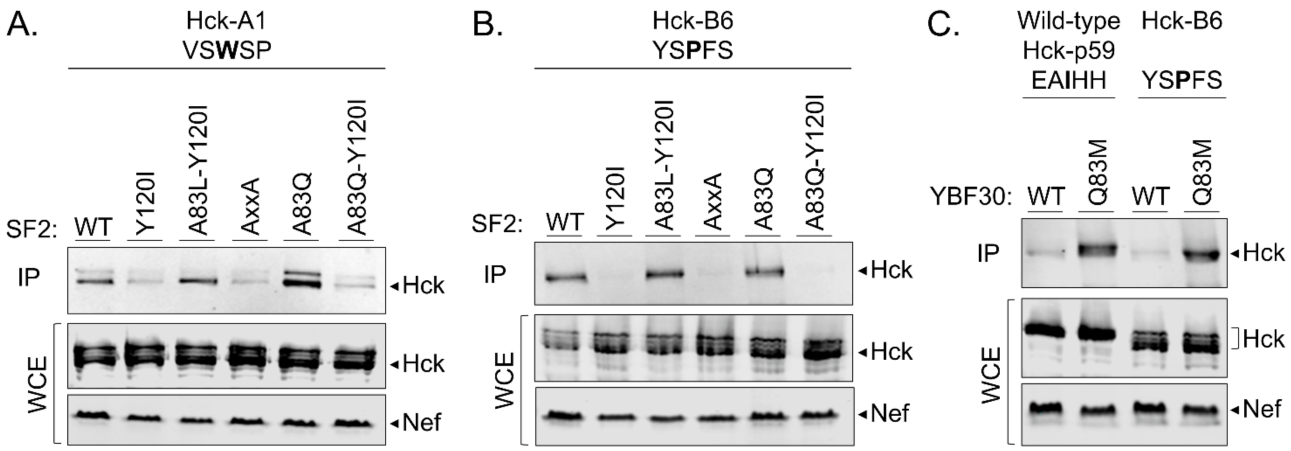

S5 Fig. Role of R-clamp residues 83/120 in intracellular Hck-Nef complex formation. HEK293 cells were transfected with Myc-tagged wild-type HIV-1 M SF2 Nef (WT) or its mutants including Y120I, A83L-Y120I, P76A+P78A (AxxA), A83Q, and A83Q-Y120I) together with biotin acceptor domain-tagged SH3 mutated Hck-p59 variants Hck-A1 (**A.**) or Hck-B6 (**B.**). Similarly, YBF30 or its R-clamp gain-of-function mutant Q83M were co-expressed with biotin acceptor domain-tagged wild-type Hck-p59 or its SH3 mutant Hck-B6. (**C.**). The Nef-contacting RT-loop residues centered around the critical Hck SH3 amino acid 13 (indicated in bold) are shown on top of these Figs. Lysates of the transfected cells were subjected to anti-Myc immunoprecipitation followed by Western blotting analysis of the immune complexes using labeled streptavidin (top panels). Equal Hck and Nef expression in the total lysates was confirmed by labeled streptavidin (Hck) (middle panels) or an anti-Myc (Nef) antibody (bottom panels).
