# Supplementary material for: Evolutionary plasticity of SH3 domain binding by Nef proteins of the HIV-1/SIVcpz lentiviral lineage
Source: PLoS Pathog. 2021 Nov 15;17(11):e1009728. doi: 10.1371/journal.ppat.1009728 (PMC8629392; doi:10.1371/journal.ppat.1009728)
Supplement: S6 Fig — (A) HEK293T cells were co-transfected with pEBB expression plasmids for the indicated Nefs and an HIV-1 reporter virus lacking functional nef and vpu genes (HIV-1 NL4-3 Δnef Δvpu IRES eGFP). Two days post transfection cell culture supernatants were harvested. Infectious virus yield was determined by infecting TZM-bl reporter cells and normalized to the amount of p24 (as determined by ELISA) to calculate virion infectivity. Mean values +/- SEM of four independent experiments are shown. (B) Two days post transfection cells were harvested for Western Blot analysis. Nef was detected via an anti-myc tag antibody. GAPDH served as loading control. (PDF) [file ppat.1009728.s006.pdf]

A.

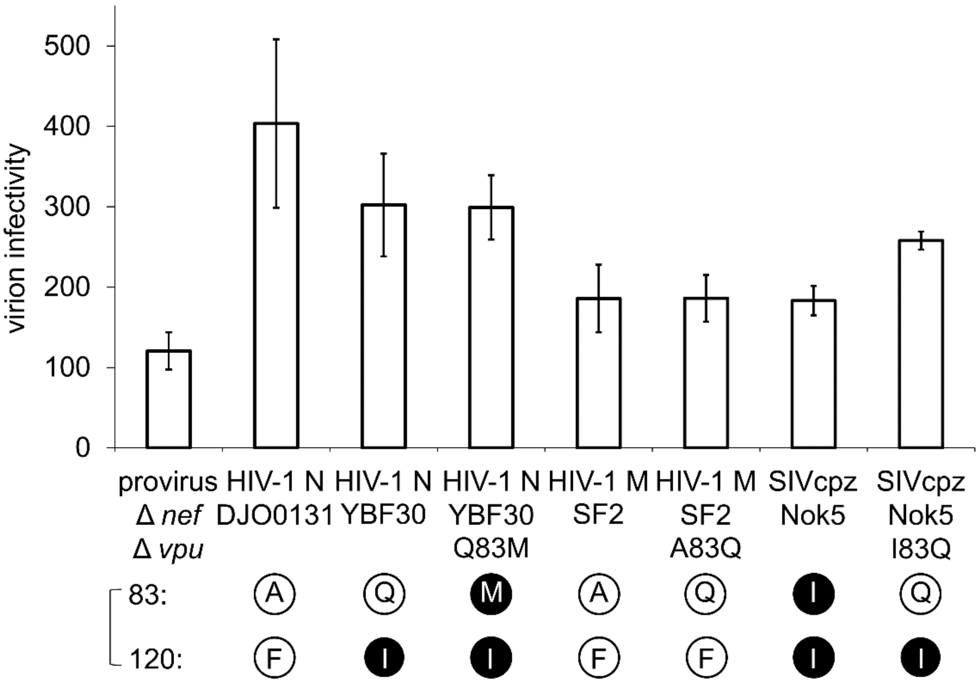

B.

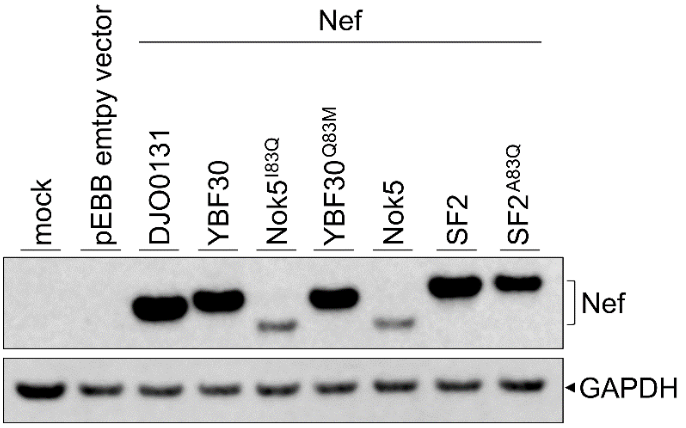

S6 Fig. Role of the R-clamp in Nef-mediated enhancement of virion infectivity. (A) HEK293T cells were co-transfected with pEBB expression plasmids for the indicated Nefs and an HIV-1 reporter virus lacking functional nef and vpu genes (HIV-1 NL4-3 Δnef Δvpu IRES eGFP). Two days post transfection cell culture supernatants were harvested. Infectious virus yield was determined by infecting TZM-bl reporter cells and normalized to the amount of p24 (as determined by ELISA) to calculate virion infectivity. Mean values +/- SEM of four independent experiments are shown. **(B)** Two days post transfection cells were harvested for Western Blot analysis. Nef was detected via an anti-myc tag antibody. GAPDH served as loading control.
