# Supplementary material for: Evolutionary plasticity of SH3 domain binding by Nef proteins of the HIV-1/SIVcpz lentiviral lineage
Source: PLoS Pathog. 2021 Nov 15;17(11):e1009728. doi: 10.1371/journal.ppat.1009728 (PMC8629392; doi:10.1371/journal.ppat.1009728)
Supplement: Attachment — Submitted filename: Rebuttal letter.docx [file ppat.1009728.s007.docx]

Major issues

**Reviewer #1**

**Issue 1** (including discussion of Issue 1 in the Summary comments by Reviewer #1)

“I do think that it is interesting and novel hat Nef’s capacity to promote paxillin phosphorylation by Hck depends (also) on a combination of two Nef residues (in position 83 and 120); and that there are Nef isolates found where this combination is such that Nef cannot longer activate Hck. However based on the data shown, I’m not convinced about their explanation for this observation, as I will explain in the following:

The correlation between physicochemical characteristics of side chains from residues that fill the same 3D space in a protein structure is of course well known (it is used, for example to establish ‘contact maps’ for proteins). It is also well known that mutations that cause gaps in a protein structure, or in a protein complex, negatively affect the stability of the protein or protein complex. Hence the loss-of-function observed by combining two insufficiently large side chains from positions 83 and 120 are expected, as they would leave a gap in the structure. However, having a combination of 83=Q/E and 120=I should fill the available space just the same, and would also allow to support (or ‘clamp’ as the authors say) R77 with a polar interaction. Interestingly, in a putative Hck SH3 complex, Q/E83 would come close to the hydrophobic Hck Ile95, which might explain that this is an unfavourable combination for Hck SH3. When introducing a larger F/Y in position 120, then the long side chain of 83 needs to bend away from Ile96 to avoid clashes, and this might result in a more favourable hydrophobic surface for Hck I95 binding as Q83 only presents parts of its aliphatic side chain region.

So my major concern is that the observations with regard to Hck binding (or their proxy of paxillin phosphorylation) are not explained by the stabilisation (clamping) of R77, but by the attractive or repulsive interactions between Hck Ile95 and Nef 83. It has been shown that some SIV Nef alleles bind weaker to Hck SH3 and stronger to SH3 from Src kinases (Src, Fyn) that have a charged/polar residue in the position of Hck Ile95 (Collette, JBC 2000). Hence, would it be possible that the ‘category III’ Nef evolved to support binding of non-Hck SH3 domains--and that the absence of Hck SH3 binding is not an issue of lacking stabilisation of R77? I think that this alternative hypothesis needs to be addressed/ruled out before the clamping mechanism, and its proposed evolutionary repercussions and conclusions, can be accepted.

E.g. 83/120 combinations that the authors termed inactive might actually present a gain of function towards another (SH3) target. This possibility (which they mention on page 26) would majorly affect their evolutionary discussion.”

“The minimum required would be to do in vitro binding tests of selected Nef representatives (at least category I and III, ideally also from the other categories) with SH3 domains from Hck, Src and Lck, for example (SH3 RT loop residues are Ile, Arg, Ser). ITC, MST, fluorescence anisotropy or other quantitative methods could be used.

Such a complementary in vitro binding assay would also be reassuring, given that the authors used “paxillin phosphorylation” as a proxy for “Nef binding to Hck SH3 domains” which might not be equivalent. E.g. how to rule out that other Src kinases are bound and activated and then phosphorylate paxillin (Src?).”

**Our response:**

To summarize, Reviewer #1 would like us to rule out an alternative possibility that instead of contributing to the R-clamp the Nef residue 83 might be more important due to contributing to the hydrophobic pocket of Nef that accommodates an isoleucine residue in the RT-loop of Hck SH3 (an interaction outside of the canonical PxxP ligand - SH3 interface previously shown by us to greatly increase the affinity of Hck for Nef (Lee et al, Cell 1996)). Specifically, Reviewer #1 suggests that instead of failing to participate in a functional R-clamp a Gln or a Glu residue at position 83 of Nef would result in a “clash” with the hydrophobic Ile96 of Hck SH3. Reviewer #1 further hypothesizes that this unfavorable situation would emerge only if Gln/Glu 83 would occur in combination with Ile120 in Nef (= category III R-clamp) but not in combination with Tyr120 (= category II R-clamp), in which case he/she speculates that the long side chain of residue 83 might “bend away from Ile96 to avoid clashes”. Reviewer #1 finds support for this idea from an earlier paper (Collette et al, JBC 2000) showing that Fyn and Src SH3 domains (which contain a charged/polar residue at the position of Hck Ile96) bind relatively better to SIV Nef. Based on the above reasoning, Reviewer #1 conceives that Nef proteins that we classify as (non-SH3 binding) category III R-clamp members might have evolved to bind non-Hck SH3 domains and suggests us to study this theory by comparing the relative affinities of Hck vs. non-Ile96-containing SH3 domains (such as Src and Lck) for different R-clamp Nef proteins.

We find this alternative model unlikely and would also like to point out that the SIVmac239 Nef used in the Collette et al paper cited by Reviewer #1 contains a Leu83/Ile120 residue combination (thus belonging to the R-clamp category IV and not III). Consequently, the differences in its SH3 binding preferences do not support the idea proposed by Reviewer #1. Nevertheless, we have carried out an extensive set of experiments to rule out the alternative possibility suggested by Reviewer #1.

Since the Nef binding affinities of Src and Lck SH3 domains are very low (>10 mM, Arold et al, Biochemistry 1998), studying these interactions in a relevant cell-based system is not a well feasible option. Instead of the approach suggested by Reviewer #1 of studying corresponding recombinantly produced proteins with advanced biophysical techniques *in vitro,* we have made use of a previously described panel of three Hck-derived artificial SH3 domains that bind strongly to Nef but differ entirely from Hck in their RT-loop sequences and hence their mode of exploiting the hydrophobic pocket of Nef (Hiipakka et al, JMB 1999). None of these SH3 domains contain an Ile residue at position 96 (but do include Asp, a charged/hydrophilic residue), and the rest of the six residues that form the hydrophobic pocket-contacting tip of the RT-loop are unique in each of them. Thus, any additional or alternative effects that the R-clamp residues 83/120 might have on Hck SH3 binding due to a possible role in the architecture of the hydrophobic pocket of Nef would unlikely be recapitulated by any of these artificial Nef-binding SH3 domains, and certainly not by the full panel of them.

We inserted these artificial SH3 domains in place of the natural SH3 domain in full length Hck-p59, and carried out co-precipitation analyses from lysates of transfected cells to compare the resulting chimeric proteins with wild-type Hckp59 in their capacity to interact with SF2 Nef, YBF30 Nef, and relevant R-clamp mutant versions of these. Wild-type Hck co-precipitated nicely with the versions of SF2 and YBF30 Nef having a functional R-clamp, but not with those containing a “broken” R-clamp, and importantly, each of the three chimeric Hck proteins carrying unique and non-Hck-like Nef-contacting RT-loops fully recapitulated this same pattern of R-clamp residue dependence in their Nef binding. These findings strongly support our original conclusion that Nef residues 83/120 are critical because of their role as components of the R-clamp to orchestrate PxxPxR motif binding, and not because of a role in accommodating the RT-loop of Hck-SH3.

Panel A of new Figure 7 shows these co-precipitation data for wild-type Hck (which also addresses the comments by Reviewer #3), and Panel B for one of these RT-loop mutated Hck proteins (C3). Similar data for the two other SH3-modified Hck proteins (A1 and B6) are shown in new Supplementary Figure 5. These experiments are discussed on pages 16-18 of the revised manuscript.

**Issue 2**

“I would also suggest to analyse the protein stability of selected Nef mutants using DSF, DSLS or CD”

**Our response:**

The steady state levels of all proteins used in our work have been examined by Western blotting. These blots are shown in the corresponding figures of the paper, and support that our conclusions are not confounded by protein stability issues. Moreover, we also provide evidence that none of the Nef mutants suffer from a generalized functional decline, as they are all active in CD4 downregulation (and according to our new data also in MHC-I downregulation and enhancement of virion infectivity; see our responses to Issues 1 and 2 raised by Reviewer #2). The biophysical techniques mentioned by Reviewer #1 are typically used to examine folding and thermal stability of purified proteins. Since we have addressed his/her first concern using intracellular co-expression of different Nef and Hck protein variants, producing recombinant proteins for such additional in vitro assays has not been pursued.

**Reviewer #2**

**Issue 1**

“A more relevant cell type should be used to test R-clamp function, such as differentiated THP-1 or primary macrophages”

**Our response:**

As suggested by Reviewer #2, we have now examined this question also in THP-1-derived human macrophages. We have examined cellular activation triggered by lentivirally transduced Nef in GM-CSF-differentiated THP-1 macrophages by monitoring Erk1/2 phosphorylation. We show a strong correlation between the capacity of the Nef variants used to induce Erk1/2 phosphorylation in these cells and the functionality of their R-clamp as defined by our previous studies in HEK293 cells. These data are now shown in new Figure 8 and discussed on pages 18-19 of the revised manuscript.

**Issue 2**

“The authors show as a control that the mutations do not affect CD4 downregulation. This is not surprising since the polyproline domain of Nef is not required for this function. Other functions dependent on a functional PxxP domain should be tested, such as MHC-I downmodulation and cell activation”

**Our response:**

As mentioned above (Issue 1), we have now studied Nef PxxP-dependent cellular activation by monitoring Erk1/2 phosphorylation, and show that this also requires a functional R-clamp (Figure 8; Results (page 18-19)).

As requested by this Reviewer, we also examined MHC-I downmodulation by Nef. However, unlike suggested by early work (Greenberg et al, EMBO J 1988), more recent studies have in fact clearly shown that despite involving proline residue 78, MHC-I downregulation is not an SH3 binding-dependent function of Nef (Fackler et al, Virology 2006; Casartelli et al, J Gen Virol 2006). Accordingly, we found that mutations in R-clamp residues 83/120 did not affect this function of Nef.

However, our manuscript already includes extensive data on CD4 downregulation, which is the best characterized SH3-independent function of Nef that also provides an assay with an excellent dynamic range. Moreover, we have now included data on enhancement of HIV-1 virion infectivity (see Issue 3 below), a Nef function which is not strongly dependent on SH3 binding (Fackler et al, Virology 2006; Shi et al. J Virol 2018) and accordingly was found not to significantly involve the R-clamp (new Supplementary Figure 6). Considering the existing length and complexity of this paper, we would therefore prefer not to include these MHC-I data as yet another SH3-independent Nef function in our study, and instead just provide these data in this rebuttal letter (see below).

**Issue 3**

“What is the impact of mutations in the R-clamp on viral infectivity?”

**Our response:**

The ability of Nef to increase viral infectivity is influenced but is not strongly dependent on an intact PxxP motif (e.g. Fackler et al, Virology 2006), and it has been shown that SH3 binding capacity of Nef is fully dispensable for SERINC5 suppression that mediates this infectivity effect (Shi et al. J Virol 2018). In this regard, the promotion of HIV infectivity by Nef is not a very informative model for studying R-clamp function. Nevertheless, we have determined the effect of a representative set of Nef protein variants on HIV-1 particle infectivity. As now shown in new Supplementary Figure 6 and mentioned on pages 19-20, mutations in R-clamp residues 83/120 did not significantly affect Nef-mediated enhancement of virion infectivity.

**Issue 4**

“A more detailed phylogenetic analysis of R-clamp evolution in various HIV/SIV groups could be helpful to understand how the authors see R-clamp selection and evolution”

**Our response:**

We have engaged a new collaborator (Kei Sato) with expertise in lentiviral evolution and carried out phylogenetic analysis of SIVcpz, SIVgor, and HIV-1 Nef proteins representing different R-clamp categories (panel B in Figure 9 [old Figure 7]). Together with previously inferred most recent common ancestors of HIV-1 M, N and O Nefs (Kluge et al, Cell Host Microbe 2014), these analyses are in line with the model presented in our Discussion, in which category I Nefs of SIVcpz P.t.t. and SIVgor gave rise to the Nef proteins of HIV-1 groups M, O and P. In contrast, HIV-1 N is most likely the result of a cross-species transmission event of an SIVcpz P.t.t. strain harboring a category III Nef that subsequently evolved into category I and IV Nefs. We have also made several minor editions (including the change in the order of the 3^rd^ and 2^nd^ last paragraphs) to our Discussion in order to improve its clarity and to better explain and justify the model on R-clamp selection during primate lentiviral evolution suggested by our data.

**Reviewer #3**

**Issue 1**

“While this assay is convincing it should be supported by a more direct evidence of the formation, or absence, of the interaction, such as using a tagged protein of one of the interactors followed by a pull-down approach to test whether the second interacting partner is indeed bound or not”

**Our response:**

As suggested, we have performed pull-down assays to study the physical interaction of wild-type and R-clamp mutated Nef proteins with Hck in transfected cells. These data provide direct evidence of the effect on R-clamp residue 83/120 composition on the ability of Nef to interact with Hck, and are in full agreement with our conclusions based on Hck kinase activation by Nef. These data are now included panel A of new Figure 7 and discussed in the text (pages 16-17).

Minor issues

**Reviewer #1**

**Issue 1**

“I found the ‘clamp’ term to be misleading. A clamp implies that an entity is held in place by forces from two opposite sides. However what the authors describe is more a one-sided support of R77. The 3D fixing of R77 is also the result of the Arg side chain with the backbone carbonyl and side chain of Q118. But R77 is not ‘clamped’ by positions 83 and 120”.

**Our response:**

We agree that Nef residues 83/120 both support the Arg77 residue of the PxxPxR motif from one side. However, together with the archetypal SH3 residue W37 (SH3 numbering according to Saksela & Permi, FEBS Lett 2012) they do form a clamp-like structure that holds the sidechain of Arg77 in place. We have discussed this issue with additional colleagues in structural biology, who also share our view that R-clamp is an appropriate and descriptive name for the set of molecular interactions that we discuss. Thus, we would like to stick to this term.

**Issue 2**

“Figure 1A: the expression levels of those sequences that do not lead to paxillin phosphorylation appear to be the lowest. If so, then this should be noted and discussed.”

**Our response:**

We agree that in the Western blot shown in Figure 1A wild-type YBF30 and the Y120I mutant of SF2 Nef seem to be expressed somewhat less than the other Nef proteins. However, this difference is modest (Y120I is 70.9% % of wild-type SF2 when the intensity of LI-COR infrared detection signal is quantified), compared to the total lack of paxillin phosphorylation and AP-1 activity. Importantly, these modestly reduced YBF30 and SF2-Y120I Nef protein levels are unique to this blot, and no correlation with R-clamp functionality and the steady-state expression levels of these or other Nef proteins were observed in the dozens of Western blots that we have run, including those shown in new Figures 7A, 7B, 8A, 8B, S5A, S5B, S5C, and S6B of the revised version of our paper.

While we therefore do not consider this point raised by Reviewer #1 to be a concern, we have modified the wording of sentences referring to the Western blot in Figure 1A to be more cautious: 1) “YBF30 Nef was expressed well in these cells” was changed into “YBF30 Nef was clearly expressed in these cells” and 2) “Y120I mutation recapitulated the failure of YBF30 Nef to stimulate paxillin phosphorylation and AP‑1 activity, without compromising protein stability or CD4 downregulation” was changed into “Y120I mutation recapitulated the failure of YBF30 Nef to stimulate paxillin phosphorylation and AP‑1 activity, without **substantially** compromising protein expression or CD4 downregulation”.

**Issue 3**

“P14: “the Y120I mutation recapitulated the failure of YBF30 Nef to stimulate paxillin phosphorylation and AP-1 activity, without compromising protein stability or CD4 downregulation (Figure 1).” Here the authors do not measure protein stability, but only CD4 downregulation. Protein stability should be measured in vitro, using DSF or DSLS or equivalent methods.”

**Our response:**

We agree that our Western blot analyses examine steady-state protein expression in the transfected cells, and not strictly speaking protein stability. As we have not performed biophysical analyses that could directly measure protein stability we have now rephrased this sentence into “without substantially compromising protein **expression levels** or CD4 downregulation”.

**Issue 4**

“Table 1: I would put ‘residue 83’ in column 2 and ‘residue 120’ in column 3, to preserve the sequence order.”

**Our response:**

Table 1 has now been rearranged as requested by Reviewer #1.

**Issue 5**

“P21: “this places R77 of Nef into close proximity with another highly conserved SH3 residue (D17) to form a salt bridge that stabilizes the Nef/SH3 complex.” I would suggest to use Hck numbering here, rather than a more arbitrary ‘SH3 numbering’.”

**Our response:**

We have used a generic SH3 numbering (Saksela & Permi, FEBS Lett 2012) for referring to the amino acid residues of Hck because this makes it easier and more explicit to discuss the corresponding key residues (such as D17) of SH3 domains from different proteins. In fact, the current discussion on the Nef-contacting “Hck SH3 residue Ile96” highlights why this important. Reviewer #1 refers to this residue as “Ile96” (or more often “Ile95”). However, to be correct this residue is Ile71 or Ile92 depending on whether the human p59 or p61 isoform of Hck is discussed. The corresponding residue in the human Fyn protein is Arg96, and the inaccurate concept of “Hck SH3 Ile96” stems from our early work on Nef binding properties of chimeric Hck/Fyn SH3 proteins (Lee et al, EMBO J, 1995). Referring to this residue using generic SH3 numbering as Fyn R13 or Hck I13 avoids all such confusion, and in our opinion is much more practical and preferable. Moreover, when using a generic SH3 numbering system, the Nef and Hck residue numbers discussed in the text and labeled in our figures do not numerically overlap, which increases clarity and reduces chances for misunderstanding. Therefore, we would like to stick with this universal SH3 numbering system.

**Issue 6**

“P22: “Why would about 25% of all HIV-1 N Nefs harbor an inactive R-clamp, whereas their counterparts from HIV-1 groups M, O, and P do not?” As long as the category III has not been shown to not bind to any SH3 domain in vitro, I don’t think that these Nefs can be catalogued as ‘inactive’.”

**Our response:**

This is an interesting question which we have already contemplated in our Discussion. It should be noted that HIV-1 group N constitutes a very small number of viruses, and has failed to effectively spread in the human population. Also, as noted in our paper, there is an apparent trend for the more recent HIV-1 N isolates to contain a functional R-clamp as compared to the earlier isolates. As SH3 binding is associated with pathogenesis of HIV-1 and SIVmac239 infection, but not required for viral replication per se, it is possible that viruses carrying an SH3 binding deficient Nef protein might be favored at some early steps during or immediately after the zoonotic jump from chimpanzees to man. On the other hand, as Reviewer #1 notes, it would be very difficult to exclude that R-clamp category III Nefs could have some affinity for one of the >300 human SH3 domains. The core SH3 ligand in Nef is a Class II binding motif ^72^PxxPxR^77^, and R-clamp-mediated coordination of its R77 residue would be expected to be relevant for all canonical SH3 domains containing the archetypical D17 and W37 residues. However, although being unable to bind Hck or other classical SH3 domains, interaction with some atypical SH3 domain can of course not be excluded. Both of the above ideas were already touched in our Discussion, but have now been more thoroughly addressed (the first paragraphs on pages 22 and 26)

**Issue 7**

“P24: in the second paragraph, two or three difference findings and probably manuscripts are all referenced as [36]. Is it possible that the first and 3rd ref 36 are actually https://doi.org/10.1016/j.jmb.2004.09.015?”

**Our response:**

We apologize for this error and thank Reviewer #1 for pointing it out. Indeed, the sentence in the Discussion mentioning Choi and Smithgall, as well as the first incorrect citation to Aldehaiman et al (old ref. #36) are supposed to cite the JMB paper by Choi & Smithgall indicated above by Reviewer #1, and only the second citation to Aldehaiman et al is correctly citing that paper, exactly as Reviewer #1 suggests. This mistake has now been corrected.

**Reviewer #2**

**No minor issues**

**Reviewer #3**

**Issue 1**

“The uthors focus on one SH3-containing protein - Hck, and only briefly mentioned other proteins that the virus interacts with through this motif. Can a more detailed discussion regarding why this mechanism of arginine R-clamp is not needed in those proteins be added, at least in Discussion (if not in additional experiments)?”

**Our response:**

As explained in our response above to Minor Issue #6 or Reviewer #1, this question has now been better addressed in our Discussion (pages 22 and 26).

**Issue 2**

“In Introduction: I suggest changing “pushing” to “shifting”: Nef tightly binds to the SH3 domain of Hck, thereby pushing it from an intramolecular autoinhibitory state into a catalytically active conformation [9, 10].”

**Our response:**

We have now changed the wording of this sentence as suggested.

References

Arold S, O'Brien R, Franken P, Strub MP, Hoh F, Dumas C, Ladbury JE. RT loop flexibility enhances the specificity of Src family SH3 domains for HIV-1 Nef. Biochemistry. 1998 Oct 20;37(42):14683-91.

Casartelli N, Giolo G, Neri F, Haller C, Potestà M, Rossi P, Fackler OT, Doria M. The Pro78 residue regulates the capacity of the human immunodeficiency virus type 1 Nef protein to inhibit recycling of major histocompatibility complex class I molecules in an SH3-independent manner. J Gen Virol. 2006 Aug;87(Pt 8):2291-2296.

Collette Y, Arold S, Picard C, Janvier K, Benichou S, Benarous R, Olive D, Dumas C. HIV-2 and SIV nef proteins target different Src family SH3 domains than does HIV-1 Nef because of a triple amino acid substitution. J Biol Chem. 2000 Feb 11;275(6):4171-6.

Fackler OT, Moris A, Tibroni N, Giese SI, Glass B, Schwartz O, Kräusslich HG. Functional characterization of HIV-1 Nef mutants in the context of viral infection. Virology. 2006 Aug 1;351(2):322-39.

Greenberg ME, Iafrate AJ, Skowronski J. The SH3 domain-binding surface and an acidic motif in HIV-1 Nef regulate trafficking of class I MHC complexes. EMBO J. 1998 May 15;17(10):2777-89.

Hiipakka M, Poikonen K, Saksela K. SH3 domains with high affinity and engineered ligand specificities targeted to HIV-1 Nef. J Mol Biol. 1999 Nov 12;293(5):1097-106

Kluge SF, Mack K, Iyer SS, Pujol FM, Heigele A, Learn GH, Usmani SM, Sauter D, Joas S, Hotter D, Bibollet-Ruche F, Plenderleith LJ, Peeters M, Geyer M, Sharp PM, Fackler OT, Hahn BH, Kirchhoff F. Nef proteins of epidemic HIV-1 group O strains antagonize human tetherin. Cell Host Microbe. 2014 Nov 12;16(5):639-50.

Lee CH, Saksela K, Mirza UA, Chait BT, Kuriyan J. Crystal structure of the conserved core of HIV-1 Nef complexed with a Src family SH3 domain. Cell. 1996 Jun 14;85(6):931-42.

Saksela K, Permi P. SH3 domain ligand binding: What's the consensus and where's the specificity? FEBS Lett. 2012;586(17):2609-14.

Shi J, Xiong R, Zhou T, Su P, Zhang X, Qiu X, Li H, Li S, Yu C, Wang B, Ding C, Smithgall TE, Zheng YH. HIV-1 Nef Antagonizes SERINC5 Restriction by Downregulation of SERINC5 via the Endosome/Lysosome System. J Virol. 2018 May 14;92(11):e00196-18.
